# Neuronal Actin Dynamics, Spine Density and Neuronal Dendritic Complexity Are Regulated by CAP2

**DOI:** 10.3389/fncel.2016.00180

**Published:** 2016-07-26

**Authors:** Atul Kumar, Lars Paeger, Kosmas Kosmas, Peter Kloppenburg, Angelika A. Noegel, Vivek S. Peche

**Affiliations:** ^1^Institute of Biochemistry I, Medical Faculty, University of Cologne, CologneGermany; ^2^Center for Molecular Medicine Cologne, University of Cologne, CologneGermany; ^3^Cologne Excellence Cluster on Cellular Stress Responses in Aging-Associated Diseases, University of Cologne, CologneGermany; ^4^Biocenter, Institute for Zoology, University of Cologne, CologneGermany

**Keywords:** actin, actin binding proteins, AMPA, CAP2, cyclase associated protein, dendritic spine, n-cofilin

## Abstract

Actin remodeling is crucial for dendritic spine development, morphology and density. CAP2 is a regulator of actin dynamics through sequestering G-actin and severing F-actin. In a mouse model, ablation of CAP2 leads to cardiovascular defects and delayed wound healing. This report investigates the role of CAP2 in the brain using *Cap2^gt/gt^* mice. Dendritic complexity, the number and morphology of dendritic spines were altered in *Cap2^gt/gt^* with increased number of excitatory synapses. This was accompanied by increased F-actin content and F-actin accumulation in cultured *Cap2^gt/gt^* neurons. Moreover, reduced surface GluA1 was observed in mutant neurons under basal condition and after induction of chemical LTP. Additionally, we show an interaction between CAP2 and n-cofilin, presumably mediated through the C-terminal domain of CAP2 and dependent on cofilin Ser3 phosphorylation. *In vivo*, the consequences of this interaction were altered phosphorylated cofilin levels and formation of cofilin aggregates in the neurons. Thus, our studies identify a novel role of CAP2 in neuronal development and neuronal actin dynamics.

## Introduction

The coordinated assembly and disassembly of the neuronal actin cytoskeleton defines the range and complexity of morphologies in neurons like dendrite formation, spine development, and synaptic plasticity (Luo, 2002; Hotulainen and Hoogenraad, 2010). Many actin regulating proteins influence morphology and plasticity of neurons. Among various actin binding proteins, cyclase associated protein (CAP) family is highly conserved and regulates actin filament dynamics. It maintains the F/G actin ratio by sequestering and severing actin. Two isoforms are present in higher eukaryotes, namely CAP1 and CAP2 (Yu et al., 1994; Swiston et al., 1995; Peche et al., 2007). CAP1 shows a wide tissue distribution whereas CAP2 is primarily present in brain, heart and skeletal muscle, skin, and testis. CAP1 has been reported as neuronal growth cone associated protein and as regulator of growth cone morphology through rearrangement of F-actin (Nozumi et al., 2009; Lu et al., 2011). Mammalian CAP1 was also shown to be a proapoptotic protein (Wang et al., 2008). CAP2 sequesters G-actin and efficiently fragments filaments. This latter activity resides in its WH2 domain (Peche et al., 2013). Earlier we reported on the function of CAP2 in the heart and in wound healing using mice lacking CAP2. Ablation of CAP2 is lethal and mice lacking CAP2 develop cardiomyopathy and have a disarrayed sarcomeric organization. Only a subset of mice survives and overcomes the lethal phenotype by an unknown mechanism (Peche et al., 2013; Field et al., 2015; Stockigt et al., 2016). During wound healing its loss leads to delayed wound closure presumably due to altered actin dynamics in keratinocytes and fibroblasts (Kosmas et al., 2015). The CAP2 gene is present at chromosome 6p22.3 in human. An interstitial 6p22-24 deletion syndrome of the short arm of chromosome 6 was reported where patients with this deletion have a variable phenotype including a developmental delay, heart defects and cognitive dysfunction. Overlapping deletions from various patients revealed the presence of Cap2 in this locus (Davies et al., 1999; Bremer et al., 2009).

Given the abundance of CAP2 in the brain, its clinical association with 6p22-24 deletion syndrome, its actin regulatory properties and restricted tissue distribution pattern, it may have a significant role in the brain and in particular for neuronal development. To directly approach the function of Cap2 in neuronal development we utilized the Cap2 mouse model. In the current study we unravel the function of CAP2 in the mutant brain and its role in regulating dendrite morphology, spine development and synaptic plasticity. We show that CAP2 is expressed in different regions of the embryonic and adult brain. Further, we analyzed the expression of CAP2 in *in vitro* neuronal cultures and found that it is expressed in soma, dendrites, pre and post synaptic terminals. Absence of Cap2 has an impact of neuronal development. In particular, dendritic complexity, the number and morphology of dendritic spines were dependent on CAP2. Furthermore, CAP2 is an important regulator of neuronal F-actin and loss of CAP2 leads to increased F-actin content. In addition, we reveal the role of CAP2 in surface trafficking of GluA1, where CAP2 loss accounts for the decrease in surface GluA1. We demonstrate that CAP2 interacts with actin filament depolymerizing protein n-cofilin through its C-terminal domain. This interaction is cofilin Ser3 phosphorylation dependent. Interestingly, *in vivo* analysis of mutant brain revealed decreased phospho-n-cofilin levels which was associated with its aberrant localization. In conclusion, these data delineate a novel role of CAP2 in neuronal development, specifically in dendritic complexity, spine density and morphology and AMPA trafficking presumably through its impact on actin and cofilin regulation.

## Results

### CAP2 Is Expressed in the Brain and Localizes to the Various Neuronal Compartments

**FIGURE 1:**
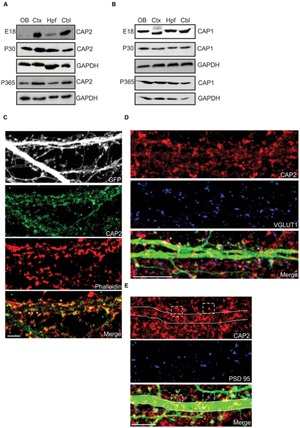
**Expression and localization of CAP2 in brain. (A)** Western blot analysis of CAP2 in lysates from dissected brain regions at different developmental stage. **(B)** Western blot analysis of CAP1 in lysates from dissected brain regions at different developmental stage. **(C)** Immunocytochemistry of cultured cortical pyramidal neurons demonstrates the presence of CAP2 (green) in the dendritic shaft and mature spine. F-actin rich structures were visualized with TRITC-phalloidin (red). Scale bar, 10 μm. **(D)** Co-labeling with antibodies against VGLUT1 (blue) revealed the presence of CAP2 in excitatory presynaptic terminal of pEGFP-C2 labeled neurons (merge, white asteriks). However, homogeneous distribution was observed in the axonal shaft. Scale bar, 10 μm. **(E)** Co-labeling of cultured cortical neurons with antibodies against CAP2 (red) and PSD-95 (blue) revealed that CAP2 localizes with PSD-95 (white dashed square). Scale bar, 5 μm.

For a detailed analysis of CAP2 expression in whole brain, we used the gene trap mice and followed the β-galactosidase fusion protein derived from the LacZ reporter and observed high expression in the olfactory bulb, cortex, hippocampus and cerebellum (**Supplementary Figure S1A**). Western blot analysis with lysates from various brain regions at E18, P30, and P365 showed that the CAP2 levels were relatively low in the olfactory bulb and hippocampus at E18 whereas at P30 and P365 the levels were increased compared to E18 (**Figure 1A**). In contrast, CAP1 was present at relatively high levels in these parts of the brain at E18. However, at P30 and P365 CAP1 was expressed uniformly in all regions of the brain (**Figure 1B**). Immunofluorescence analysis revealed CAP2 in the cortex, hippocampus and cerebellum (**Supplementary Figure S1B**).

To analyze the neuronal localization of CAP2, we performed immunofluorescence analysis with cultured cortical pyramidal neurons, which revealed a homogeneous distribution of CAP2 throughout the cytosol and in neurites. Using TRITC-phalloidin to label F-actin, we concluded that CAP2 colocalizes with F-actin (**Figure 1C**, white arrow; 65.1 ± 12.9% colocalization with CAP2; 5–6 neurons each from 3 different cultures). CAP2 also localizes in the dendritic shaft and presynaptic terminal as revealed by MAP2 and synapsin I staining in neurons (**Supplementary Figures S1C,D**). Colocalization experiments with vGLUT1 suggested that CAP2 colocalizes in excitatory presynaptic terminals (**Figure 1D**, white asterisks; 54.3 ± 8.7% colocalization with CAP2; 5–6 neurons each from 3 different cultures). In addition to this, CAP2 also colocalizes with PSD-95 in postsynaptic terminals (**Figure 1E**, white square; 51.6 ± 13.0% colocalization with CAP2; 5–6 neurons each from 3 different cultures).

Therefore, it is possible that CAP2 might influence the dendritic morphology, dendritic protrusions and spine development. Given the importance of actin as the most prominent cytoskeletal protein at both the pre- and post-synaptic terminals and our data showing the distribution of CAP2 in the pre and post synaptic matrix, it might well be that CAP2 plays an important role in synaptic processes.

### Altered Dendritic Complexity and Spine Density in CAP2 Mutant Neurons

**FIGURE 2:**
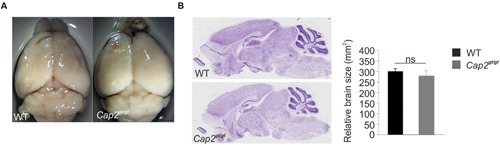
**Morphology and anatomy of *Cap2^gt/gt^* brain. (A)** Comparison of brains isolated from wild-type as well as from homozygous *Cap2^gt/gt^* mice does not reveal differences in the size of the brain (upper panel). Scale bar, 1 mm. **(B)** Nissl staining of sagittal sections and its graphical analysis from control and mutant brains (middle panel) does not show any significant difference in gross anatomy of brain WT: 300 ± 13 mm^2^, *n* = 10 sections from 3 mice; *Cap2^gt/gt^*: 280 ± 22 mm^2^, *n* = 10 sections from 3 mice, *p* > 0.05. Scale bar, 250 μm.

We confirmed the complete deletion of CAP2 at the protein level in mutant mice by western blot and immunofluorescence analysis as earlier reported (**Supplementary Figures S2A,B**; Peche et al., 2013). Although CAP2 is expressed in the WT brain, no obvious alterations in the brain of *Cap2^gt/gt^* mice with regard to size and overall morphology were noted (**Figures 2A,B**).

**FIGURE 3:**
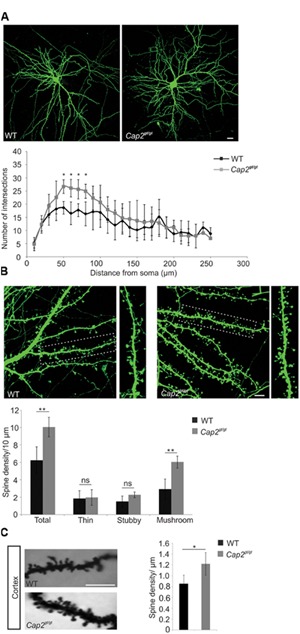
**Neuronal dendritic complexity and spine density is altered in mutant neurons. (A)** An increase in dendritic complexity and dendritic spine was observed at div 16 in cultured cortical neurons labeled with pEGFP-C2. Sholl analysis revealed the number of dendrite intersections of cortical neurons (Scale bar, 20 μm, *p* < 0.05). **(B)** Primary cortical neurons from WT and CAP2 mutant mice transfected with pEGFP-C2 show an increased linear density of dendritic spine in mutant neurons (WT: 6.3 ± 1.5 spines/10 μm, *n* = 20 neurons from 3 different cultures; *Cap2^gt/gt^*: 10.1 ± 1.1 spines/10 μm, *n* = 15 neurons from 3 different cultures, *p* < 0.01) and of mushroom-like dendritic spines (WT: 2.9 ± 1.2 spines/10 μm, *n* = 20 neurons from 3 different cultures; *Cap2^gt/gt^*: 6.0 ± 0.7 spines/10 μm, *n* = 15 neurons from 3 different cultures, *p* < 0.01). Stubby-like (WT: 1.5 ± 0.6 spines/10 μm, *n* = 20 neurons from 3 different cultures; *Cap2^gt/gt^*: 2.3 ± 0.3 spines/10 μm, 15 neurons from 3 different cultures, *p* > 0.05) and Thin-like (WT: 1.8 ± 0.9 spines/10 μm, *n* = 20 neurons from 3 different cultures; *Cap2^gt/gt^*: 2.0 ± 0.9 spines/10 μm, *n* = 15 neurons from 3 different cultures, *p* > 0.05) dendritic spines were not altered. Scale bar, 20 μm. **(C)** Golgi cox staining in WT and CAP2 mutant cortex and quantification revealed an increase in spine density (WT: 0.85 ± 0.17 spine/μm; *Cap2^gt/gt^*: 1.2 ± 0.2 spine/μm, *n* = 20 neurons from 3 different mice, *p* < 0.05). Scale bar, 20 μm. *p < 0.05, **p < 0.01.

CAP2 is present in the neuronal dendrites where actin is predominant and the degree of actin polymerization plays a crucial role in maintaining spine density and dendritic complexity (Hotulainen and Hoogenraad, 2010). To analyze the function of CAP2 in spine development and morphogenesis, we used cultured primary cortical neurons, transfected with pEGFP-C2 and observed an increase in dendritic complexity in CAP2 mutant neurons (**Figure 3A**). Quantitative analysis using the Sholl method revealed a statistically significant increase in dendritic arbor complexity at a distance of 50–80 μm from the soma in the CAP2 mutant neurons (**Figure 3A**, *p* < 0.05). This suggests an increased dendritic bifurcation, which is a determinant of dendritic length. Next we analyzed the dendritic spine development at day *in vitro* (div) 7 and observed an increase in dendritic protrusions in mutant neurons (**Supplementary Figure S2C**; WT: 0.3 ± 0.05 dendritic protrusions/μm, *n* = 30 neurons from 3 different cultures; *Cap2^gt/gt^*: 0.54 ± 0.04 dendritic protrusions/μm, *n* = 24 neurons from 3 different cultures, *p* < 0.01). Further analysis of cultured cortical neurons at div 16 showed an effect of deletion of CAP2 on dendritic spine development and maturation. Qualitative analysis suggests an increase in dendritic spines in mutant neurons (**Figure 3B**). We therefore quantified the number of dendritic spines using NeuronStudio, which revealed an increase in spine density in CAP2 null neurons (**Figure 3B**; WT: 6.3 ± 1.5 spines/10 μm, *n* = 20 neurons from 3 different cultures; *Cap2^gt/gt^*: 10.1 ± 1.1 spines/10 μm, *n* = 15 neurons from 3 different cultures, *p* < 0.01). Furthermore, classification of spine morphologies revealed that CAP2 mutant neurons have more mushroom-shaped spines compared to WT neurons. However, there was no difference observed in stubby-shaped and thin spines (**Figure 3B**; mushroom-like spines WT: 2.9 ± 1.2 spines/10 μm, *n* = 20 neurons from 3 different cultures; *Cap2^gt/gt^*: 6.0 ± 0.7 spines/10 μm, *n* = 15 neurons from 3 different cultures, *p* < 0.01; stubby-like spines WT: 1.5 ± 0.6 spines/10 μm, *n* = 20 neurons from 3 different cultures; *Cap2^gt/gt^*: 2.3 ± 0.3 spines/10 μm, *n* = 15 neurons from 3 different cultures, *p* > 0.05; thin-like spines WT: 1.8 ± 0.9 spines/10 μm, *n* = 20 neurons from 3 different cultures; *Cap2^gt/gt^*: 2.0 ± 0.9 spines/10 μm, *n* = 15 neurons from 3 different cultures, *p* > 0.05). Along the same line, Golgi-Cox staining of the cortex revealed a significant increase in spine density in *Cap2^gt/gt^* neurons (**Figure 3C**; WT: 0.85 ± 0.17 spines/μm; *n* = 20 neurons from 3 mice; *Cap2^gt/gt^*: 1.2 ± 0.2 spines/μm; *n* = 20 neurons from 3 mice, *p* < 0.05). Additionally visualization of hippocampal neurons through Golgi-Cox staining revealed a similar trend of increased spine density in *Cap2^gt/gt^* neurons (**Supplementary Figure S2D**). Taken together, our data highlight a role of CAP2 in dendritic complexity and dendritic spine density.

**FIGURE 4:**
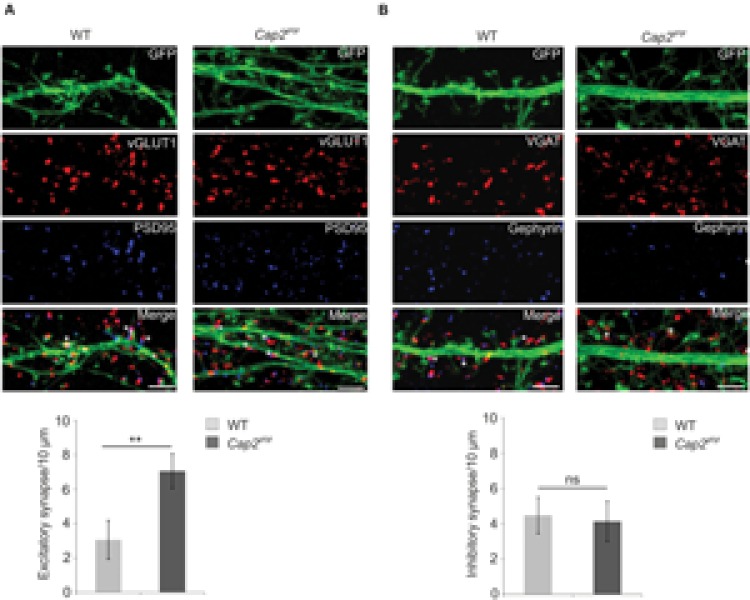
**(A)** pEGFP-C2 transfected primary cortical neurons at div 16 labeled with antibodies against vGLUT1 (a presynaptic marker, red) and PSD-95 (a postsynaptic marker, blue). An increase in excitatory synapse (white asterik) was observed in CAP2 mutant neurons (WT: 3.1 ± 1.1 synapses/10 μm, *n* = 20 neurons from 4 experiments; *Cap2^gt/gt^*: 7.1 ± 1.0 synapses/10 μm, *n* = 20 neurons from 3 experiments, *p* < 0.01). Scale bar, 5 μm. **(B)** pEGFP-C2 transfected primary cortical neurons at div 16 labeled with antibodies against VGAT (an inhibitory presynaptic marker, red) and gephyrin (an inhibitory postsynaptic marker, blue). There is no significant difference in inhibitory synapses (white asterisk) in CAP2 mutant neurons (WT: 4.5 ± 1.0 synapses/10 μm, *n* = 20 neurons from 4 experiments; *Cap2^gt/gt^*: 4.1 ± 1.1 synapses/10 μm, *n* = 20 neurons from 3 experiments, *p* > 0.05). Scale bar, 5 μm. **p < 0.01.

To analyze synapse development, we studied the ability of *in vitro* cultured neurons to form excitatory or inhibitory synapses. We labeled the neurons with antibodies against PSD-95 and vGLUT1, gephyrin and vGAT, markers for excitatory and inhibitory synapses, respectively. PSD-95 and vGLUT1colocalizing puncta denotes the developed excitatory synapse while puncta positive for gephyrin and vGAT signifies the inhibitory synapse. We observed a significant increase in excitatory synapses in CAP2 knockout cortical neurons in comparison to the control (**Figure 4A**, WT: 3.1 ± 1.1 synapses/ 10 μm, *n* = 20 neurons from 4 experiments; *Cap2^gt/gt^*: 7.1 ± 1.0 synapses/ 10 μm, *n* = 20 neurons from 3 experiments, *p* < 0.01 asterisks indicate puncta positive for VGLUT1 and PSD-95). However, labeling of neurons with gephyrin and vGAT which are markers for inhibitory postsynaptic and presynaptic terminal did not reveal any significant difference in inhibitory synapses in CAP2 knockout neurons (**Figure 4B**, WT: 4.5 ± 1.0 synapses/10 μm, *n* = 20 neurons from 4 experiments; *Cap2^gt/gt^*: 4.1 ± 1.1 synapses/10 μm, *n* = 20 neurons from 3 experiments, *p* > 0.05; asterisks indicate puncta positive for gephyrin and vGAT). This result suggests that CAP2 regulates the dendritic spine density with a concomitant change in the density of excitatory synapses.

### Altered Dendritic Complexity in CAP2 Mutant Purkinje Neurons

**FIGURE 5:**
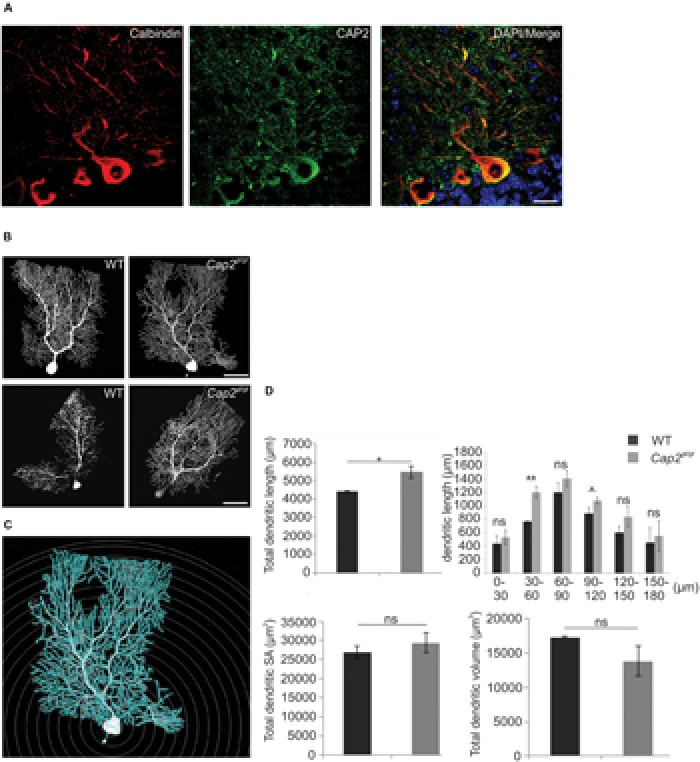
**Dendritic complexity is altered in mutant Purkinje neurons. (A)** CAP2 (green) shows diffuse distribution in the cytoplasm and dendrites of Purkinje neurons labeled with antibodies against calbindin (red). Scale bar 20 μm. **(B)** Purkinje neuron in cerebellum was injected with fluorescent dye biocytin at P60 (Upper panel) and P365 (lower panel) to evaluate neuronal complexity and morphology in CAP2 mutant mice. **(C)** Sholl analysis using NeuronStudio was performed for P60. Scale bar, 20 μm. **(D)** Quantification of Sholl analysis for Purkinje neurons revealed an increase in total dendritic length but not in dendritic surface area and dendritic volume. *p < 0.05, **p < 0.01.

We analyzed Purkinje neurons, which show high structural complexity when compared to many other neuron types. Expression of CAP2 in the external granule layer and Purkinje layer of cerebellar folia was a potential indicator of a possible role of CAP2 in Purkinje neurons (**Supplementary Figure S1B**). We co-stained brain sections with polyclonal CAP2 antibodies and monoclonal calbindin antibodies, a Purkinje neuronal marker. Calbindin is a 28 kDa vitamin D-dependent Ca^2+^ ion-binding protein. It stains cell bodies as well as processes of the specific neuronal subpopulation hence it is useful in analyzing neuronal morphology. The Purkinje layer contains the cell bodies of the Purkinje neurons. It extends its dendritic tree into the molecular layer where it forms synaptic connections with other neurons of the cerebellum like granule neurons, stellate cells and basket cells (Carletti and Rossi, 2007). Strong staining of CAP2 was observed in the cytoplasm and dendritic arborization of the Purkinje neuron (**Figure 5A**).

We next labeled individual Purkinje neurons in the cerebellum with fluorescent dye biocytin/streptavidin at P60 and P365, followed by microscopic visualization. Confocal images revealed an increased dendritic complexity in mutant Purkinje neurons as compared to the WT (**Figure 5B**). Furthermore, we performed Sholl analysis on stacked confocal images using NeuronStudio for P60 (**Figure 5C**). Purkinje neurons from WT mice had a total dendritic length of ~4430 μm whereas CAP2 mutant mice showed an increase in dendritic length to ~5470 μm (**Figure 5D**; WT: 4430 ± 30 μm, *n* = 7 neurons; *Cap2^gt/gt^*: 5470 ± 340 μm, *n* = 9 neurons; *p* < 0.05). The difference in dendritic length was more prominent in basal (WT: 770 ± 10 μm, *n* = 7 neurons; *Cap2^gt/gt^*: 1200 ± 90 μm, *n* = 9 neurons; *p* < 0.01) and apo-basal regions (WT: 890 ± 90 μm, *n* = 7 neurons; *Cap2^gt/gt^*: 1080 ± 50 μm, *n* = 9 neurons; *p* < 0.05) than in the apical region of Purkinje neurons. However, we did not observe a significant difference in the total dendritic surface area (**Figure 5D**; WT: 26910 ± 1620 μm^2^, *n* = 7 neurons; *Cap2^gt/gt^*: 29410 ± 2600 μm^2^, *n* = 9 neurons; *p* > 0.05) and total dendritic volume (**Figure 5D**; WT: 17300 ± 150 μm^3^, *n* = 7 neurons; *Cap2^gt/gt^*: 13820 ± 2230 μm^3^; *n* = 9 neurons; *p* > 0.05) of CAP2 mutant Purkinje neurons.

### Actin Filament Turnover Is Impaired in CAP2 Deficient Neurons

**FIGURE 6:**
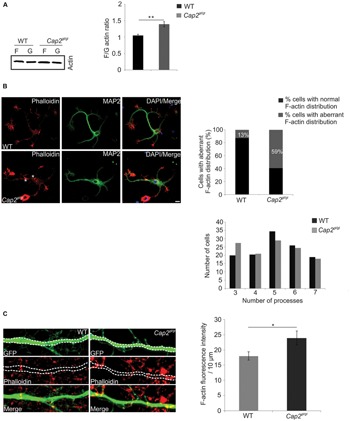
**CAP2 deletion leads to actin accumulation and impaired actin dynamics. (A)** Immunoblotting of WT and CAP2 mutant brain lysate for determination of the F/G-actin ratio revealed an increase in F-actin in mutant brain lysate (WT: 1.06 ± 0.03, *n* = 4 mice; *Cap2^gt/gt^*: 1.3 ± 0.1, *n* = 4 mice, *p* < 0.01). **(B)** Immunocytochemistry of cultured cortical neurons after 24 h in cultures demonstrated F-actin accumulation in the soma and in neuritic processes of mutant neurons (white asteriks). F-actin was visualized with phalloidin staining. Scale bar, 10 μm. Quantification of WT and mutant neurons showing aberrant F-actin distribution in the soma and/or neuritic processes. Quantification of dendritic processes. The distribution of cells with a certain number of processes is shown. **(C)** pEGFP-C2 transfected neurons labeled with TRITC-phallodin at div 16. Quantification revealed a significant increase in F-actin intensity in the dendritic shaft (white dashed line; WT: 18.0 ± 1.3 F-actin intensity (AU)/10 μm; *Cap2^gt/gt^*: 24.0 ± 2.3 F-actin intensity (AU)/10 μm, *n* = 20 neurons from 3 experiments, *p* < 0.05). Scale bar, 5 μm. AU, arbitrary unit; *p < 0.05, **p < 0.01.

The effects on dendritic complexity indicated CAP2 as an essential component in regulating actin dynamics during the maturation of neurons. To examine this possibility directly, we tested the F/G actin ratio in whole brain lysates from CAP2 mutant mice. We lysed forebrain tissue and performed fractionation assays to separate F-actin and G-actin. Western blot quantification revealed a 1.3-fold increase in F/G actin ratio in the CAP2 mutant brain lysate when compared to WT indicating an increase of F-actin in the mutant brain lysates (**Figure 6A**, WT: 1.06 ± 0.03, *n* = 4 mice; *Cap2^gt/gt^*: 1.3 ± 0.1, *n* = 4 mice, *p* < 0.01).

In neurons, F-actin is primarily enriched at dendritic spines and forms a complex network which supports the structures of dendritic spines (Cohen et al., 1985). Our initial observation of increased F/G actin ratio in mutant brain prompted us to analyze the F-actin distribution microscopically in *in vitro* cultured neurons. In WT neurons, F-actin rich structures, detected with phalloidin staining, were predominantly present at the neurite growth cone. MAP2 was used as dendritic marker (**Figure 6B**). Interestingly, unlike WT neurons, F-actin was accumulated in the soma as well as in the neuritic processes of mutant neurons (**Figure 6B**, asterisk). We further counted cells with aberrant actin rich structures and found it to be statistically significant (**Figure 6B**, WT: 13 ± 3.7% cells with aberrant F-actin, *n* = 225 cells from 3 preparations; *Cap2^gt/gt^*: 59 ± 2.6% cells with aberrant F-actin, *n* = 225 cells from 3 preparations, *p* < 0.01). The alteration in the F-actin distribution prompted us to analyze neurite outgrowth. We could not observe a difference in the initial spreading of WT and mutant neurons within the first 24 h after plating as evident by the unaltered average number of processes per cell (**Figure 6B**). We next labeled pEGFP-C2 transfected neurons with TRITC-phalloidin to examine F-actin in neurons (**Figure 6C**). We quantified the F-actin intensity per 10 μm of neuronal dendrite and observed an increase in F-actin (**Figure 6C**; WT: 18.00 ± 1.3 F-actin intensity (AU)/10 μm; *Cap2^gt/gt^*: 24.00 ± 2.3 F-actin intensity (AU)/10 μm, *n* = 20 neurons from 3 preparations, *p* < 0.05). To investigate a possible CAP1 compensatory effect in the brain, we checked the expression of CAP1 in WT and *Cap2^gt/gt^* mice brain lysates. Immunoblotting with CAP1 polyclonal antibodies did not reveal any difference in the brain CAP1 levels (**Supplementary Figure S2E**; WT: 3.0 ± 1.03 AU, *n* = 5 mice; *Cap2^gt/gt^*: 3.15 ± 1.05 AU, *n* = 6 mice; *p* > 0.05).

### CAP2 Knockout Cultured Cortical Neurons Show Reduced Density of Surface Dendritic GluA1

**FIGURE 7:**
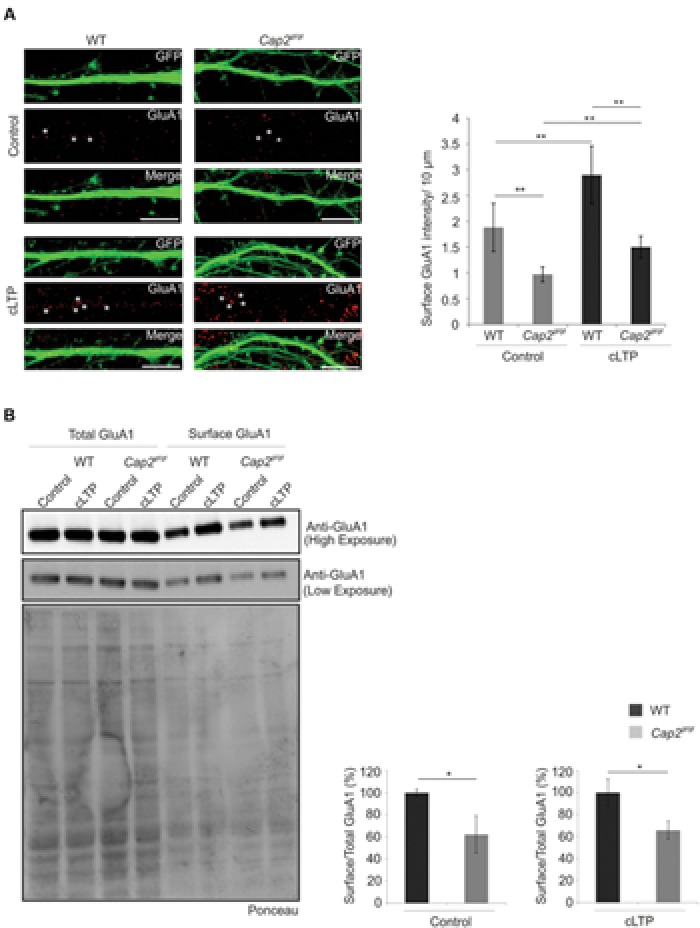
**Surface AMPA receptor trafficking is impaired in CAP2 null neurons. (A)** pEGFP-C2 transfected WT and CAP2 mutant neurons labeled with antibodies against GluA1 in control and upon cLTP induction. Scale bar, 10 μm. Quantification of data revealed a significant decrease in GluA1 surface intensity in control condition and upon cLTP induction. (WT control: 1.8 ± 0.46 surface GluA1 intensity (AU)/10 μm; *Cap2^gt/gt^* control: 0.97 ± 0.14 surface GluA1 intensity (AU)/10 μm; white asterisk; *n* = 40 neurons from 3 set of different experiments; *p* < 0.01; WT cLTP: 2.9 ± 0.55 surface GluA1 intensity (AU)/10 μm; *Cap2^gt/gt^* cLTP: 1.5 ± 0.2 surface GluA1 intensity (AU)/10 μm; white arrows; *n* = 40 neurons from 3 set of different experiments; *p* < 0.01). **(B)** Surface biotinylation and western blotting with GluA1 in control and cLTP induced cortical neurons. Quantification of data revealed a significant decrease in surface GluA1 in CAP2 mutant neurons in control condition (WT control surface/total: 100 ± 3.2%; *Cap2^gt/gt^* control surface/total: 62.3 ± 16.8%; *n* = 3 neuronal preparations; *p* < 0.05) and upon cLTP induction (WT cLTP surface/total: 100 ± 12.1%; *Cap2^gt/gt^* cLTP surface/total: 65.9 ± 8.3%; *n* = 3 neuronal preparations; *p* < 0.05). Ponceau staining of the nitrocellulose membrane as loading control for the pull down assay of surface AMPA. *p < 0.05, **p < 0.01.

The actin cytoskeleton plays an important role during synaptic receptor trafficking during plasticity. AMPA receptors traffic constitutively to and from the plasma membrane via recycling endosomes. AMPA receptor trafficking occurs constitutively under basal condition and is modulated by activity through changes in actin and myosin dynamics (Bredt and Nicoll, 2003; Rocca et al., 2008). CAP2 is widely expressed in the dendritic shaft of neurons which makes it important to examine its role in receptor trafficking. To examine the effect of CAP2 on the surface AMPA receptor, we utilized chemically induce long term potentiation (cLTP). We labeled GluA1, a subunit of the AMPA receptor at div 16 under control condition and glycine induced cLTP. In control condition, *Cap2^gt/gt^* neurons show reduced surface density of GluA1 compared to the surface GluA1 in WT cortical neurons. However, upon induction of cLTP there is a significant increase in surface GluA1 in both WT and CAP2 knockout neurons. This increase of surface GluA1 in mutant neurons upon cLTP is significantly less compared to the WT neurons, which suggest synaptic plasticity is impaired in CAP2 mutant neurons (**Figure 7A**, WT control: 1.8 ± 0.46 surface GluA1 intensity (AU)/10 μm; *Cap2^gt/gt^* control: 0.97 ± 0.14 surface GluA1 intensity (AU)/10 μm; *n* = 40 neurons from 3 different preparations, **p < 0.01; WT cLTP: 2.9 ± 0.55 surface GluA1 intensity (AU)/10 μm; *Cap2^gt/gt^* cLTP: 1.5 ± 0.2 surface GluA1 intensity (AU)/10 μm; *n* = 40 neurons from 3 different preparations, *p* < 0.01; white asterisks indicate surface GluA1). This was further confirmed by surface biotinylation assays, which revealed that surface GluA1 levels under basal and cLTP condition were reduced in CAP2 knockout neurons compared to WT neurons while total GluA1 was unchanged (**Figure 7B**; WT control surface/total: 100 ± 3.2%; *Cap2^gt/gt^* control surface/total: 62.3 ± 16.8%, *n* = 3 neuronal preparations, *p* < 0.05; WT cLTP surface/total: 100 ± 12.1%; *Cap2^gt/gt^* cLTP surface/total: 65.9 ± 8.3%, *n* = 3 neuronal preparations, *p* < 0.05). This suggests that CAP2 is important for the exocytosis of AMPA receptor.

### CAP2 Interacts with n-Cofilin

**FIGURE 8:**
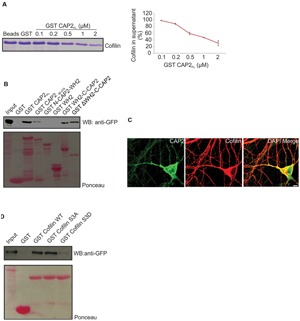
**CAP2 interacts with n-cofilin through its C-terminal domain. (A)** Coomassie-stained SDS-polyacrylamide gel for the supernatant depletion pull down assay for examining the interaction between the GST-CAP2 and thrombin cleaved cofilin protein from GST-cofilin protein. The amount of cofilin in the supernatant fractions was quantified from Coomassie-stained SDS-polyacrylamide gels by densitometry analysis using Image J (GST: 1; 0.1μM GST-CAP2_FL_: 0.98 ± 0.01; 0.2 μM GST-CAP2_FL_: 0.88 ± 0.02; 0.5 μM GST-CAP2_FL_: 0.59 ± 0.04; 1 μM GST-CAP2_FL_: 0.47 ± 0.01; 2 μM GST-CAP2_FL_: 0.3 ± 0.07; *n* =3). **(B)** Pull-down experiment with ectopically expressed pEGFP-C2 n-cofilin. Precipitated proteins were detected by western blotting using monoclonal anti-GFP. Ponceau staining of the nitrocellulose membrane as loading control for the pull down assay. GST was used as a control. CAP2 interacts with n-cofilin through its C terminal. **(C)** Immunocytochemical analysis by co-labeling with monoclonal CAP2 (green) and polyclonal cofilin I (red) revealed the colocalization in adult brain section. **(D)** Immunoblot of pull down using bacterially expressed purified GST-n-cofilin WT, GST-n-cofilin S3A mutant showed a pull down of CAP2 from the lysates whereas GST-n-cofilin S3D which mimics a phosphorylated Ser3 residue had reduced binding efficiency for CAP2. Ponceau staining as loading control of nitrocellulose membrane for pull down assay. GST was used as a control.

Previous studies reported that CAP accelerates the dissociation of ADP G-actin-cofilin complexes, recharges actin monomers with ATP and releases cofilin from the ATP G-actin complex for actin polymerization (Moriyama and Yahara, 2002). CAP1 interacts with cofilin in muscle and non-muscle cells. The interaction between CAP2 and cofilin has not been addressed so far. We performed supernatant depletion pull down assays with variable concentrations of bead-immobilized GST-CAP2 and purified n-cofilin. A decrease of n-cofilin in the supernatant was observed which shows an interaction between CAP2 and n-cofilin. The assay was carried out at physiological pH and salt concentration (**Figure 8A**). Next we narrowed down the interacting domain of CAP2 by performing pull down experiments with ectopically expressed pEGFP-C2-n-cofilin and GST-tagged CAP2 polypeptides (Peche et al., 2013). We observed a strong interaction of n-cofilin and C-CAP2 which was presumably WH2 domain independent. N terminal and WH2 domain did not show any interaction whereas a weak interaction was observed with a truncated protein lacking the first 55 amino acids of CAP2 (**Figure 8B**). This points at the importance of the C terminal domain of CAP2 for the interaction with n-cofilin. The CAP2-n-cofilin interaction was also supported by colocalization studies in WT primary cortical neurons where we found partial colocalization of n-cofilin and CAP2 at the cell periphery as well as in the cytoplasm and neurites (**Figure 8C**).

Cofilin exists in a phosphorylated and an unphosphorylated state, the latter being the active state (Sarmiere and Bamburg, 2004). Hence to analyze whether CAP2 binds to the phosphorylated (inactive) or unphosphorylated (active) n-cofilin, we used mutant proteins carrying mutations of serine 3 mimicking the phosphorylated (S3D) or unphosphorylated state (S3A) of cofilin. GST-WT n-cofilin and GST-n-cofilin WT S3A mutant pulled down CAP2 from lysates of pEGFP-C2-CAP2 expressing HEK293T cells, whereas GST-n-cofilin S3D showed very weak binding to CAP2 (**Figure 8D**). Thus CAP2 has a potential to interact with n-cofilin and the activation state of cofilin also has an effect on this interaction.

### CAP2 Affects the Phosphorylated n-cofilin Levels and Leads to Its Aggregation

**FIGURE 9:**
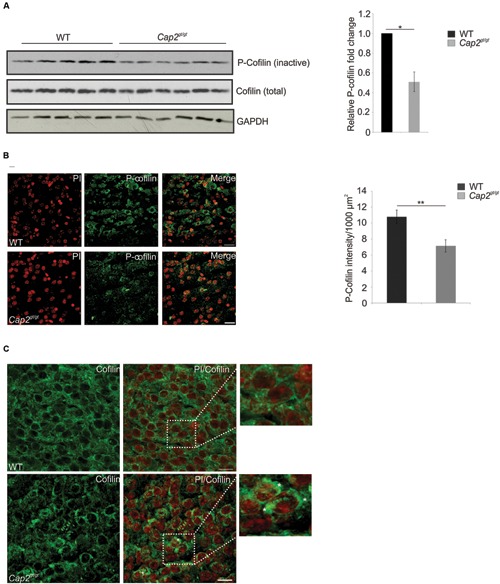
**CAP2 depletion increases cofilin dephosphorylation and its accumulation. (A)** Western blotting of phospho Ser3 cofilin shows reduced cofilin phosphorylation in the CAP2 mutant cortical lysate. Total cofilin expression was unchanged in WT and mutant cortical lysate. GAPDH served as a loading control. Quantification of cofilin phosphorylation from six independent experiments analyzed using Student’s *t*-test (WT: 1; *Cap2^gt/gt^*: 0.5 ± 0.1; *n* = 6, *p* < 0.05). Values were normalized to the loading control. **(B)** Immunohistochemical analysis of WT and CAP2 mutant brain section with rabbit monoclonal phospho cofilin antibody revealed a decrease in staining in the cortical region of mutant brain. Scale bar, 10 μM. Quantification of phospho-cofilin revealed a significant difference in mutant brain (WT: 10.8 ± 0.8 P-n-cofilin intensity (AU)/1000 μm^2^; *Cap2^gt/gt^*: 7.2 ± 0.8 P-n-cofilin intensity (AU)/1000 μm^2^; 2–4 sections per mice, total 4 mice, *p* < 0.01). **(C)** WT and *Cap2^gt/gt^* brain sections were stained with rabbit monoclonal cofilin antibodies. Accumulation of cofilin in cytoplasmic aggregates was observed in CAP2 mutant brain as indicated by white asterisks, whereas cofilin was nicely distributed in the cytoplasm and nucleus of WT brain. Scale bar, 10 μM. *p < 0.05, **p < 0.01.

The CAP2-n-cofilin interaction directed us to examine the effect of this interaction on cofilin regulation in neurons. The levels of phosphorylated n-cofilin were lower in cortical brain lysates of *Cap2^gt/gt^* mice compared to WT (**Figure 9A**; WT: 1; *Cap2^gt/gt^*: 0.5 ± 0.1 relative fold change to WT; *n* = 6; *p* < 0.05). Supporting this observation, immunofluorescence analysis also revealed low levels of phospho (Ser3) cofilin in the mutant brain (**Figure 9B**; WT: 10.8 ± 0.8 P-n-cofilin intensity (AU)/1000 μm^2^; *Cap2^gt/gt^*: 7.2 ± 0.8 P-n-cofilin intensity (AU)/1000 μm^2^; *n* = 2–4 sections/mice from 4 different mice per genotype; *p* < 0.01). The total n-cofilin content was unaltered (**Figure 9A**). We next examined a possible alteration in total n-cofilin localization in CAP2 mutant brain. In WT, n-cofilin was diffusively present in the cytosol whereas in mutant brain, we found n-cofilin accumulation in cytoplasmic aggregates, where it is presumably inactive (**Figure 9C**, asterisk). We also analyzed the expression of kinases and phosphatases involved in n-cofilin phosphorylation and dephosphorylation. Western blotting of phospho LIMK1/LIMK2 and LIMK2 did not show significant differences in their amounts in WT and CAP2 mutant brain lysates. The expression of TESK1 and ROCK1, kinases involved in n-cofilin phosphorylation, were also unaltered. Moreover, the levels of the phosphatase chronophin remained unchanged (**Supplementary Figure S3**). Taken together these results confirm that ablation of CAP2 leads to increased levels of dephosphorylated n-cofilin along with n-cofilin aggregation.

## Discussion

The actin cytoskeleton plays a major role in the morphology and structural changes of neurons. The equilibrium between G- and F-actin regulates many cellular processes and the pool of G- and F-actin is maintained by many actin binding proteins. CAP2 is a G-actin sequestering protein and also shows a F-actin severing potential, making it an important regulator of actin dynamics (Peche et al., 2007, 2013). We reported previously that CAP2 knockout mice develop dilated cardiomyopathy (Peche et al., 2013), furthermore, it plays a role in skin repair during wound healing, and its ablation leads to changes in infiltration of inflammatory cells and contraction of wounds (Kosmas et al., 2015).

In mature neurons, AMPA receptors are delivered to the plasma membrane by exocytosis at extrasynaptic sites in the soma or dendritic shaft and travel to the dendritic spine via lateral diffusion (Patterson et al., 2010; Anggono and Huganir, 2012). The number of AMPA receptors at the synaptic and extrasynaptic site of the plasma membrane depends on relative rates of exocytosis and endocytosis. CAP2 has been reported to interact with ADAM 10 a metalloproteinase and a role for CAP2 in the localization of ADAM 10 in neurons and its activity toward amyloid precursor protein (APP) has been proposed (Pelucchi et al. unpublished data; Abstract, 9th FENS Forum of Neuroscience 2014). ADAM 10 is localized in the postsynaptic density region of the excitatory synapse and regulates its activity via N-cadherin cleavage (Malinverno et al., 2010; Gardoni et al., 2012). N-cadherin activation results in more surface AMPA receptors which result in bigger and stable synapses (Kasai et al., 2003; Xie et al., 2008). Decreased surface ADAM 10 has been reported during LTP in hippocampal neurons while upon long term depression an increase surface ADAM 10 has been observed (Marcello et al., 2013). Therefore it brings in CAP2 as a downstream effector in regulating surface AMPA during neuronal plasticity.

An increase in F-actin was observed in lysates from CAP2 mutant brain. F-actin staining in cultured WT neurons showed F-actin rich structures predominantly at the neuritic growth cone, whereas actin punctae were observed in the soma and neuritic processes of mutant neurons. Depletion of CAP1 also leads to a heavy accumulation of abnormal F-actin structures in NIH/3T3, N2A and HeLa cells (Bertling et al., 2004; Zhang et al., 2013). F-actin aggresomes have been reported in neuronal cells which comprise an aberrant accumulation of multiple fragments of F-actin trapped in the cytoplasm (Lázaro-Diéguez et al., 2008). Similar punctae were also observed in CAP2 mutant neurons, which points at the role of CAP2 in maintaining cellular F/G actin ratios. The imbalance in this ratio could ultimately lead to the formation of these structures, which are detrimental to the cell depending on their composition and abundance.

Neuronal dendrites develop a small-specialized actin rich structure known as dendritic spine. At the structural level, more stable dendritic spines are developed from the highly motile dendritic filopodia-like structures. Actin nucleation by Arp2/3 and the formin mDia2 together with actin filament disassembly by ADF/cofilin promote localized actin filament turnover. This generates a mechanical force against the dendritic plasma membrane that can lead to initiation and elongation of dendritic filopodia (Honkura et al., 2008; Hotulainen et al., 2009). In CAP2 mutant neurons, we observed a significant increase in dendritic filopodia formation and dendritic spine density at div 7 and 16, respectively, as revealed by Sholl analysis of cortical neurons. The density of mushroom-like spines was also increased in CAP2 mutant neurons. Furthermore, Golgi Cox staining in WT and *Cap2^gt/gt^* adult brain revealed a significant increase in dendritic spines in mutants. Similar observations were made when n-cofilin was deleted from principal neurons of the postnatal forebrain, which resulted in an increased synaptic F-actin content, increased spine density and enlargement of dendritic spines (Rust et al., 2010). In various studies it has been shown that dendritic spines are also eliminated by spine pruning. Recent report suggests that the actin binding protein drebrin interacts with spikar and that this interaction controls retraction of existing spines and addition of new spines (Yamazaki et al., 2014). *Cap2^gt/gt^* neurons exhibit increased spine density. This could be coupled with our observation of CAP2 in neuronal dendrites and CAP2 being a regulator of actin filament dynamics, a role of CAP2 in dendritic filopodia formation and maintenance of dendritic spines is likely. Additionally, in CAP2 mutant Purkinje neurons we observed a difference in dendritic complexity. Although we did not observe a compensatory effect of CAP1 upon CAP2 deletion, this does not rule out the possibility of alternative mechanisms even by unchanged levels of CAP1 by which the severity of the Purkinje neuron phenotype is attenuated. Similar observations were also made in the protein family of phosphatidylinositol 3,4,5- triphosphate dependent Rac exchanger 1 (P-Rex1) and P-Rex2. A double knock out of P-Rex1 and P-Rex2 showed a very strong Purkinje cell dendritic morphology phenotype and motor neuron coordination defects in comparison to the mild phenotype of the P-Rex2 knockout (Donald et al., 2008). Very little is known about the molecular mechanism behind the interstitial branching in dendrites. However, it is thought that destabilization of cortical actin and the membrane can lead to filopodial protrusions, which act as precursors of transient branches.

Cyclase associated protein actively cooperates with cofilin and actin to regulate actin dynamics (Hubberstey and Mottillo, 2002; Moriyama and Yahara, 2002; Ono, 2013; Zhang et al., 2013). The nematode *Caenorhabditis elegans* also has two CAP isoforms; CAS-1 and CAS-2 where CAS-1 binds to actin monomers and enhances exchange of actin-bound ATP/ADP even in the presence of UNC-60B, a muscle-specific ADF/cofilin; CAS-2 strongly enhances the exchange of actin bound nucleotide in the presence of UNC-60A, a *C*. *elegans* non-muscle-specific ADF/cofilin (Nomura et al., 2012; Nomura and Ono, 2013). Our data demonstrates that CAP2 also interacts with n-cofilin. Moreover this interaction, unlike CAP1, resides in the C-terminal domain of CAP2. CAP1 and CAP2 primarily differ in their N-terminal domain, which could be one possible explanation for CAP2’s C-terminal interaction with n-cofilin. Interestingly, CAS-2 N-terminal does not interact with G-actin-UNC-60A complex suggesting it to behave similar to mouse CAP2 (Nomura and Ono, 2013). We also report that ablation of CAP2 in neuron results in the reduction of phosphorylated cofilin and its accumulation in cytoplasmic aggregates. However, reduced expression of various kinases like LIMK, TESK or ROCK was not observed in CAP2 mutant neurons. This is in agreement with earlier reports where CAP1 when knocked down gave rise to reduced cofilin phosphorylation and cofilin aggregation (Bertling et al., 2004; Zhang et al., 2013).

Many psychiatric and neurological disorders like autism spectrum disorder (ASD), schizophrenia and Alzheimer’s disease are accompanied by alterations in spine number and dendritic complexity (Penzes et al., 2011; Shirao and González-Billault, 2013). Altered neuronal actin dynamics lead to a behavioral phenotype in mice (Rust and Maritzen, 2015). In independent studies conducted at the international mouse phenotyping consortium (IMPC), Cap2 heterozygous animals show decreased rearing, implying a role of CAP2 in altering the ability to initiate locomotor activity. Furthermore, rota rod analysis of Cap2 heterozygous animals at the IMPC also revealed impaired proprioception measured through rotarod tests^1^. Further investigations with a brain specific CAP2 knockout must be done in order to conclude the brain specific function of CAP2 in the behavioral phenotypes.

n-cofilin deletion from principal neurons of the postnatal forebrain impairs synaptic plasticity during associative learning (Rust et al., 2010). Additionally, double mutant mice lacking ADF and n-cofilin, ADF^-/-^/n-Cof^flx/flx,CamKII-cre^, were shown to develop attention-deficit hyperactivity disorder (ADHD). The proposed hypothesis for the development of an ADHD-like phenotype in these mice involves impaired actin dynamics resulting in altered neurotransmitter release (Zimmermann et al., 2015).

Cyclase associated protein 2 is a gene of interest for possibly contributing to both the cognitive delay and the heart defects in patients with a chromosome 6p22 deletion (Bremer et al., 2009). Further, a number of genes involved in dopamine receptor mediated signaling pathways were dysregulated in schizophrenics. These include genes in the protein kinase A pathway (protein kinase ARII subunit, adenylyl cyclase-associated protein 2) and protein kinase C (Hakak et al., 2001). Also, upregulation of CAP1 has been reported in the brain of schizophrenic patients (Martins-de-Souza et al., 2010). This point towards a role of CAPs in neurological disorders. Therefore it will be interesting to decipher the role of CAP2 in various human neurological disorders. Further studies with human samples are needed to find possible mutations in CAP2 to support these findings.

## Conclusion

Depletion of CAP2 leads to an increase of F-actin in CAP2 mutant neurons along with an increase in dephosphorylation of cofilin and the appearance of cytoplasmic cofilin aggregates. The interaction of CAP2 and n-cofilin could be essential to keep n-cofilin in its unaggregated state thereby protecting neurons from pathological consequences. Increased F-actin levels in mutant neurons is the most probable cause of the morphological defects observed which include increased dendritic spine density and neuronal complexity. Thus, CAP2 is an important actin regulator in the brain and its deletion leads to several pathological consequences that are also found in neurological disorders.

## Materials and Methods

### Transgenic Animals

Cyclase associated protein 2 deficient mice generated by a gene trap approach have been described previously (Peche et al., 2013). C57/Bl6 wild type (WT) mice derived from the breeding of *Cap2^gt/+^* animals were used as controls.

The animals had free access to water and standard laboratory chow diet and were kept at an artificial light/dark cycle at 20–22°C. All procedures were performed in accordance with the animal protection law stated in the German civil code and were conform to the Guide for the Care and Use of Laboratory Animals published by the US National Institutes of Health (NIH Publication No. 85-23, revised 1985).

### Primary Cortical Neuronal Culture

For primary neuronal cultures, cortical neurons were dissected from E18 to E19 mouse embryos, dissociated and plated on 12 mm poly-_D_-lysine-coated coverslips at a density of 30,000–50,000 cells. Neuronal cultures were maintained in Neurobasal Medium (Invitrogen) supplemented with B-27 (Invitrogen) and 2 mM GlutaMAX (Invitrogen). Neurons were transfected with pEGFP-C2 (Clontech) using the Amaxa Neuron transfection kit (Lonza). Transfected neurons were fixed with 4% paraformaldehyde/4% sucrose in PBS for 15 min at room temperature (RT). Neurons were incubated for 1 h with primary antibody followed by washing for surface staining. For any intracellular labeling neurons were permeabilized with 0.1% Triton X-100. Images were acquired with a 63× oil immersion objective Leica SP5 confocal microscope (Leica, Germany). In colocalization experiments punctae positive for CAP2 were analyzed. Sholl analysis was performed using the NIH ImageJ Sholl Analysis Plugin (v1.0) downloaded from the Ghosh lab website^2^. Dendritic spine density and classification was performed using NeuronStudio (Beta) as reported earlier (Rodriguez et al., 2008). Excitatory synapses and inhibitory synapses were labeled with vGLUT1/PSD95 and VGAT/Gephyrin antibodies for presynaptic and postsynaptic terminals, respectively. Punctae positive for pre and postsynaptic terminals were counted per 10 μm of dendritic length. Quantification of F-actin intensity was done with Leica LAS AF lite software. Fluorescence intensity per 10 μm dendritic shaft was measured at div 16. Dendritic spines were excluded from the analysis.

### Immunohistochemistry, Antibodies, and Histology

Primary antibodies used in the studies were: anti-CAP2 polyclonal (1:100 IF; 1:800 WB), anti-CAP2 monoclonal (K82-381-1, IF: undiluted hybridoma supernatant; Kosmas et al., 2015), anti-CAP1 polyclonal (1:1200 WB; Peche et al., 2007), anti-β actin monoclonal (1:2500; WB Sigma), anti-GFP monoclonal (Blau-Wasser et al., 2009), anti-cofilin (D3F9) rabbit monoclonal (1: 100 IF; 1:1000 WB; Cell Signaling), anti-MAP2 polyclonal (1:50 IF; NEB), anti- Synapsin 1 (1:1000 IF; Thermo Scientific), anti-VGLUT1 Gp polyclonal (1:200 IF; Millipore), anti-PSD-95 monoclonal (7E3-1B8, 1:200 IF; Thermo Scientific), anti VGAT polyclonal (1:200 IF; Millipore), anti gephyrin monoclonal (1:50 IF), anti Calbindin monoclonal (1:1000; Sigma); anti GluA1 polyclonal (1:200 IF; Alomone labs; 1:1000 WB; Cell signaling), anti-phospho cofilin (Ser3) rabbit monoclonal (1:100 IF; 1:750 WB; Cell Signaling), anti-LIMK2 rabbit monoclonal (1:750 WB; Cell Signaling), anti-phospho-LIMK1 (Thr508)/LIMK2 (Thr505) rabbit monoclonal (1:750 WB; Cell signaling), anti-TESK1 rabbit monoclonal (1:1000 WB; Cell Signaling), anti ROCK1 rabbit monoclonal (1:1000 WB; Cell Signaling), anti-chronophin rabbit monoclonal (1:1000 WB; Cell Signaling). TRITC phalloidin (1:100 IF) was used to label F-actin in neurons. For western blot, HRP-conjugated anti mouse and anti-rabbit secondary antibodies (1:10,000) were used for detection. Monoclonal antibodies against GAPDH conjugated with horseradish peroxidase (1:10000 WB, Sigma, St. Louis, MO, USA) were used for analyzing equal loading. Densitometric analysis of western blot was done using Image J.

LacZ staining and immunofluorescence with paraffin sections were done as described previously (Peche et al., 2013). For brain sections incubation was done with primary antibodies. Appropriate secondary antibodies conjugated with Alexa Fluor 488 and 568 (Molecular Probes) were used. Nuclei were visualized with either 40,60-Diamidino-20-phenylindole (DAPI) or propidium iodide (PI). Sections were mounted and imaged with a Leica SP5 confocal microscope. Golgi-Cox staining (Glaser and Van Der Loos, 1981) was performed as per the manufacturer’s instructions using FD Rapid Golgi Stain kit (FD Neurotechnologies). For spine analysis, secondary and tertiary dendritic shaft were imaged from cortical regions. Dendritic spine analysis was carried out using Reconstruct software (Risher et al., 2014). Nissl staining was performed as described previously (Pilati et al., 2008). The brain size was quantified using ImageJ.

### Surface Biotinylation Assay

Surface biotinylation assay has been described previously (Smith et al., 2014). Briefly div 12–14 cortical neurons cultured on 25 mm coverslips in six well plates were incubated on ice with biotin solution [EZ-Link Sulfo-NHS-biotin (Thermo Scientific) at 0.5 mg/ml in PBS containing Ca^2+^/Mg^2+^] which were quenched further with quench buffer (PBS Ca^2+^/Mg^2+^ containing 1 mg/ml BSA). Neuronal lysates were prepared by incubating them in RIPA buffer (50 mM Tris-HCl, pH 7.5, 1 mM EDTA, 2 mM EGTA, 150 mM NaCl, 1% NP40, 0.5% DOC, 0.1% SDS and protease inhibitor cocktail). A part of supernatant was collected as a total protein sample while remainder was incubated for 2 h with 25 μl NeutrAvidin Agarose resin (Thermo Scientific) 50% slurry at 4°C to precipitate biotin labeled membrane protein. Beads were washed thrice with RIPA buffer followed by protein sample analyzed by SDS-PAGE and western blotting.

### Induction of Chemical LTP

Chemical LTP was induced as described previously (Fortin et al., 2010). Briefly, cortical neurons at div 16 were maintained in normal extracellular solution (ECS; 125 mM NaCl, 2.5 mM KCl, 2 mM CaCl_2_, 1 mM MgCl_2_, 5 mM HEPES, 30 mM Glucose; osmolarity 290 mOsm/l). LTP was induced by changing the medium to ECS supplemented with (0.2 mM glycine, 0.02 mM bicuculline, and 0.003 mM strychnine) for 10 min at room temperature. Neurons were incubated further in normal ECS for 45 min at 37°C. Surface AMPA receptors intensity (AU) per 10 μm of dendritic shaft was quantified using Leica LAF lite software.

### Western Blot

Tissue extracts from different regions of the brain at different age were prepared by homogenizing fresh tissue in ice cold lysis buffer (1% Triton X-100, 0.1 M NaCl, 0.05 M Tris-HCl pH 7.5, 0.01 M EDTA, add fresh protease inhibitor cocktail) using a tight fitting Douncer. For phospho cofilin, LIMK and phospho LIMK blot protein was isolated from E18 brain cortices as described previously (Garvalov et al., 2007). The protein concentration in the lysate was determined by using the Lowry method.

### Determination of the F/G-actin Ratio

The F/G-actin ratio determination was performed as described earlier (Lopes et al., 1999; Rust et al., 2010). Freshly dissected brain tissue from P30 mice was lysed in ice cold 1xPHEM extraction buffer (60 mM PIPES, 20 mM HEPES, 10 mM EGTA, 2 mM MgCl_2_, and 1% Triton X-100; pH 7.0) by using a tight fitting Dounce homogenizer. F-actin was pelleted by a 10 min centrifugation at 10,000 rpm. Supernatant and pellet were brought to equal volume by 1x SDS sample buffer, heated at 95°C and equal amounts of each sample were separated on 10% polyacrylamide SDS gels, transferred to nitrocellulose membranes, and subjected to immunolabeling. Anti-β actin was used as primary antibody (1:2500; Sigma), and HRP-conjugated anti mouse secondary antibody (1:10000) was used for detection. Densitometric analysis was done using Image J for estimation of the F/G-actin ratio.

### Analysis of Purkinje Cell Morphology

The animals were anesthetized with halothane (B4388; Sigma–Aldrich, Taufkirchen, Germany) and subsequently decapitated. Coronal slices (300 μm) were cut with a vibration microtome (HM-650 V; Thermo Scientific, Walldorf, Germany) under cold (4°C), carbogenated (95% O_2_ and 5% CO_2_), glycerol-based modified artificial cerebrospinal fluid (GaCSF; Ye et al., 2006) to enhance the viability of neurons. GaCSF contained 250 mM Glycerol, 2.5 mM KCl, 2 mM MgCl_2_, 2 mM CaCl_2_, 1.2 mM NaH_2_PO_4_, 10 mM HEPES, 21 mM NaHCO_3_, 5 mM Glucose and was adjusted to pH 7.2 with NaOH resulting in an osmolarity of 310 mOsm. Brain slices were transferred into carbogenated artificial cerebrospinal fluid (aCSF). First, they were kept for 20 min. in a 35°C ‘recovery bath’ and then stored at room temperature (24°C) for at least 30 min. Neurons were visualized with a fixed stage upright microscope (BX51WI, Olympus, Hamburg, Germany) using 40 and 60× water-immersion objectives (LUMplan FL/N 40×, 0.8 numerical aperture, 2 mm working distance; Olympus) with infrared differential interference contrast optics and fluorescence optics (Dodt and Zieglgansberger, 1990). One percentage Biocytin and tetramethylrhodamine-dextran was allowed to diffuse into the single cell until dendritic arborizations of Purkinje cells were clearly visible. The brain slices were fixed in Roti-Histofix (P0873; Carl Roth, Karlsruhe, Germany) overnight at 4°C and rinsed in 0.1 M PBS afterward and subsequently incubated in Alexa Fluor 633 (Alexa 633)-conjugated streptavidin (1:600; 2 h; RT; S21375; Invitrogen, Karlsruhe, Germany) that was dissolved in PBS containing 10% normal goat serum. Images were obtained using a Zeiss LSM 510 confocal laser scanning microscope with an Achroplan 40x/0.75 oil immersion objective (Zeiss). A stack of optical sections (1 μm) completely covering the labeled cell was acquired. The stacks were reassembled and the morphometric analyses were performed with ImageJ. A concentric Sholl analysis and reconstruction of neuronal structure was performed with NeuronStudio (Beta) as reported earlier (Wearne et al., 2005; Rodriguez et al., 2008). At least four animals were used for each data set.

### Pull-Down Assay

The supernatant depletion pull down assay for examining the interaction between GST-tagged CAP2 (Peche et al., 2013) and thrombin released cofilin from GST-cofilin was carried out as described (Mattila et al., 2004). The amount of cofilin in the supernatant was quantified from Coomassie-Blue stained SDS-polyacrylamide gels by densitometry using Image J. GST tagged CAP2 polypeptides were used as described previously (Peche et al., 2013) to narrow down the interacting domain of CAP2. HEK-293T cells were transfected with pEGFP-C2 cofilin (Clontech) using Amaxa Neuron transfection kit (Lonza). Pull down assays for pEGFP-C2-CAP2 and GST-cofilin (WT, S3A mutant and the S3D mutant) were performed as described previously (Zhang et al., 2013).

### Statistical Analysis

Sets of data are presented by their mean values and standard error of the means. An unpaired two tailed Student’s *t*-test was used when comparing two sets of data.

## Author Contributions

AK designed and performed experiments, carried out data analysis, and wrote the manuscript. KK performed experiments and provided reagents. LP and PK were involved in experimental design, carried out experiments and analyzed the data. VP carried out experiments. AN and VP were responsible for conception and design, data analysis and manuscript writing.

## Conflict of Interest Statement

The authors declare that the research was conducted in the absence of any commercial or financial relationships that could be construed as a potential conflict of interest.

## Footnotes

1 http://www.mousephenotype.org/data/genes/MGI:1914502

2 http://labs.biology.ucsd.edu/ghosh/software/

## Supplementary Material

FIGURE S1 **(A)** X-gal staining for *Cap2^+/gt^* brain shows a widespread distribution of CAP2. The major anatomical regions of brain olfactory bulb (OB), cortex (Ctx), hippocampus (Hpf), and cerebellum (Cbl) are depicted. Scale bar, 1 mm. **(B)** CAP2 is expressed in cortex, hippocampus and cerebellum. Paraffin embedded brain sections were stained with CAP2 monoclonal antibodies (K82-381-1) and a widespread expression profile was observed in mice brain (Scale bar, 20 μm). **(C)** CAP2 is expressed in the neuronal dendritic shaft as revealed by coimmunostaining with MAP2. **(D)** Cortical neurons were labeled with antibodies against CAP2 and synapsin I, a presynaptic marker, revealing the presence of CAP2 at presynaptic terminals (white arrows in inset).

FIGURE S2 **(A)** Western blot analysis for brain lysate with polyclonal CAP2 antibody. **(B)** Immunofluorescence analysis with brain section from WT and CAP2 mutant mice revealed complete deletion of CAP2 from brain (Scale bar, 20 μm). **(C)** Primary cortical neurons from WT and CAP2 mutant mice transfected with pEGFP-C2 showed an increased number of dendritic filopodia in mutant neurons at div 7 (WT: 0.3 ± 0.05 dendritic protrusions/μm, *n* = 30 neurons from 3 cultures; *Cap2^gt/gt^*: 0.54 ± 0.04 dendritic protrusions/μm, *n* = 24 neurons from 3 cultures, *p* < 0.01). Scale bar, 5 μm. **(D)** Representative image of second order dendritic shaft from hippocampal neurons visualized with Golgi-Cox staining. **(E)** Expression level of CAP1 in brain lysate was quantified after immunoblotting the WT and CAP2 mutant brain lysate with CAP1 polyclonal antibody (WT: 3.0 ± 1.0 AU, *n* = 5 mice; *Cap2^gt/gt^*: 3.2 ± 1.1 AU, *n* = 6 mice, *p* > 0.05). AU, arbitrary unit.

FIGURE S3 **Expression of kinases and phosphatases involved in Cofilin phosphorylation/dephosphorylation cycle**. Western blotting of phospho LIMK1/LIMK2 and LIMK2 do not show any significant difference in WT and CAP2 mutant brain lysate. The levels of other kinases like TESK1 and ROCK1 were also unaltered in mutant brain lysate as was the level of chronophin.
