# Supplementary figures and images for: Neuronal Actin Dynamics, Spine Density and Neuronal Dendritic Complexity Are Regulated by CAP2
Source: Front Cell Neurosci. 2016 Jul 26;10:180. doi: 10.3389/fncel.2016.00180 (PMC4960234; doi:10.3389/fncel.2016.00180)

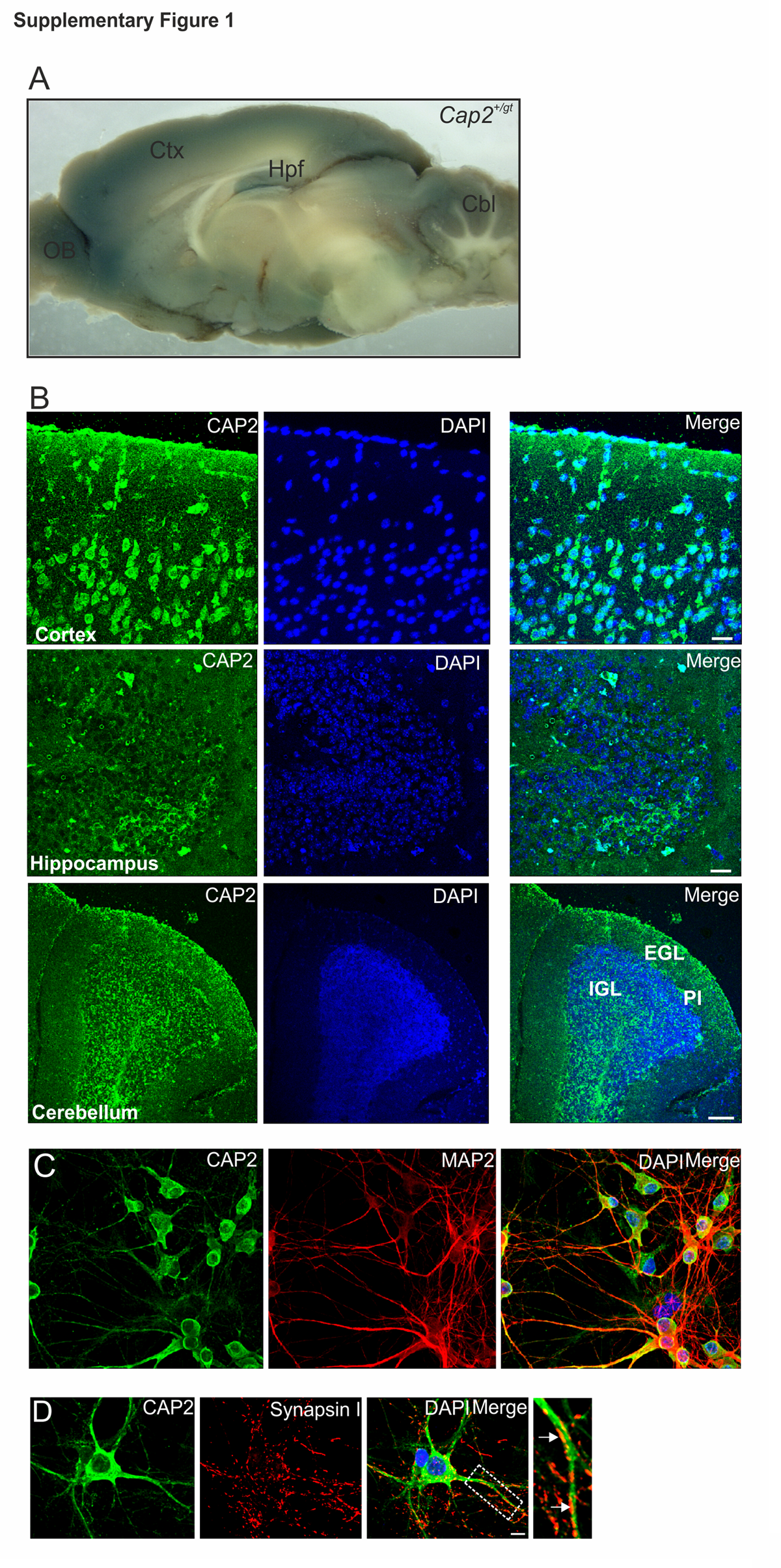

Supplement: FIGURE S1 — (A) X-gal staining for Cap2+/gt brain shows a widespread distribution of CAP2. The major anatomical regions of brain olfactory bulb (OB), cortex (Ctx), hippocampus (Hpf), and cerebellum (Cbl) are depicted. Scale bar, 1 mm. (B) CAP2 is expressed in cortex, hippocampus and cerebellum. Paraffin embedded brain sections were stained with CAP2 monoclonal antibodies (K82-381-1) and a widespread expression profile was observed in mice brain (Scale bar, 20 μm). (C) CAP2 is expressed in the neuronal dendritic shaft as revealed by coimmunostaining with MAP2. (D) Cortical neurons were labeled with antibodies against CAP2 and synapsin I, a presynaptic marker, revealing the presence of CAP2 at presynaptic terminals (white arrows in inset). [file Image_1.TIF]

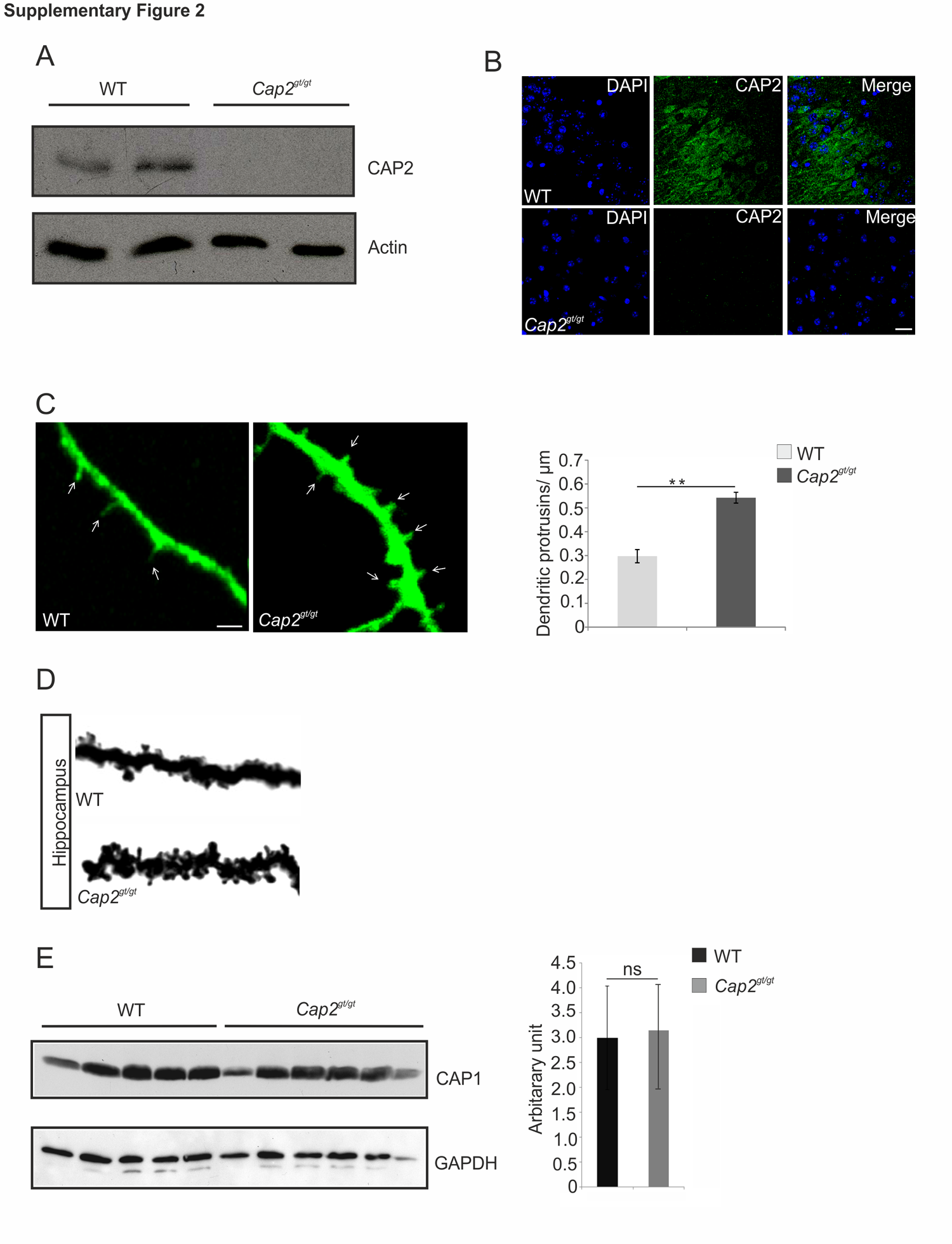

Supplement: FIGURE S2 — (A) Western blot analysis for brain lysate with polyclonal CAP2 antibody. (B) Immunofluorescence analysis with brain section from WT and CAP2 mutant mice revealed complete deletion of CAP2 from brain (Scale bar, 20 μm). (C) Primary cortical neurons from WT and CAP2 mutant mice transfected with pEGFP-C2 showed an increased number of dendritic filopodia in mutant neurons at div 7 (WT: 0.3 ± 0.05 dendritic protrusions/μm, n = 30 neurons from 3 cultures; Cap2gt/gt: 0.54 ± 0.04 dendritic protrusions/μm, n = 24 neurons from 3 cultures, p < 0.01). Scale bar, 5 μm. (D) Representative image of second order dendritic shaft from hippocampal neurons visualized with Golgi-Cox staining. (E) Expression level of CAP1 in brain lysate was quantified after immunoblotting the WT and CAP2 mutant brain lysate with CAP1 polyclonal antibody (WT: 3.0 ± 1.0 AU, n = 5 mice; Cap2gt/gt: 3.2 ± 1.1 AU, n = 6 mice, p > 0.05). AU, arbitrary unit. [file Image_2.TIF]

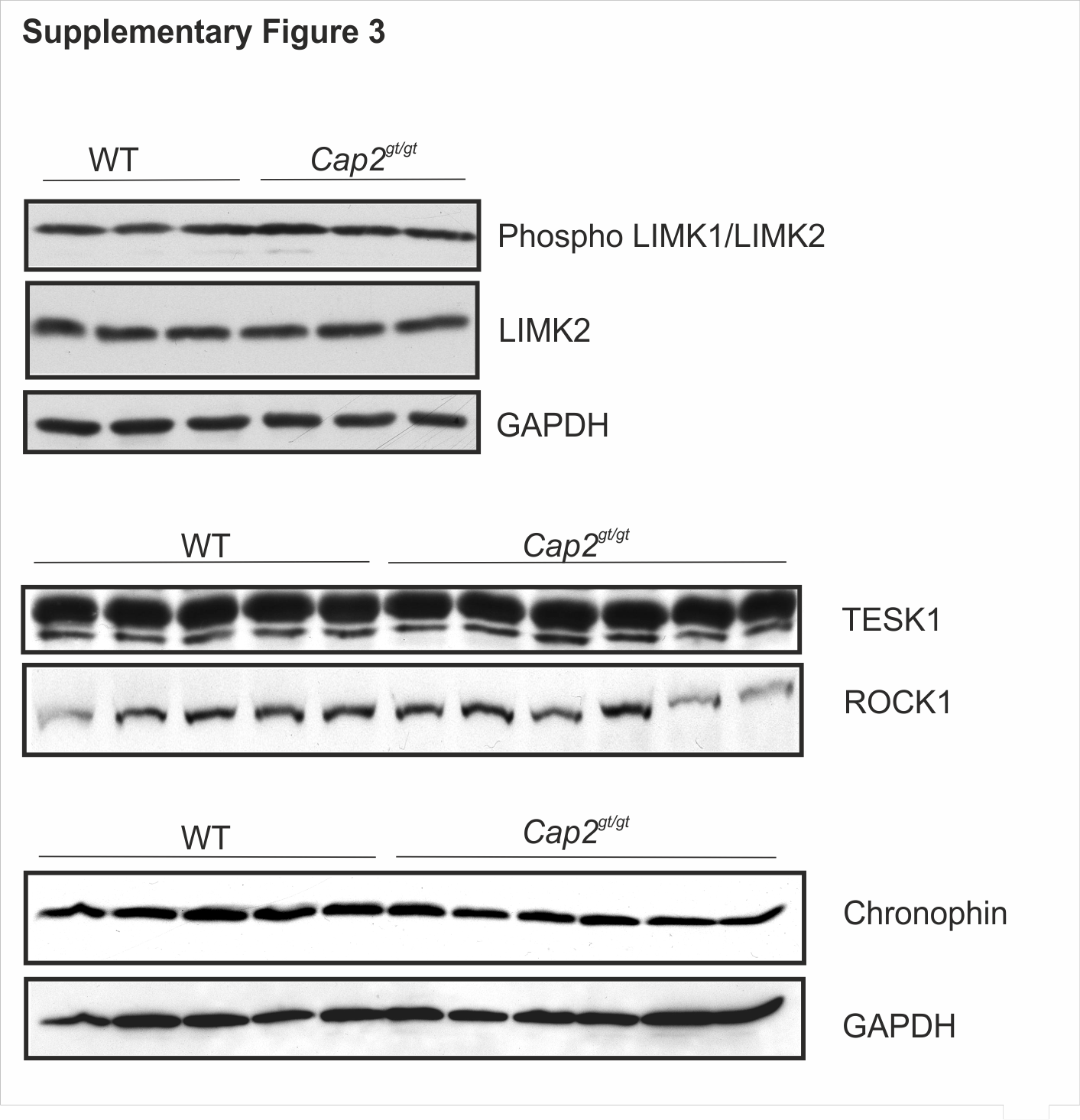

Supplement: FIGURE S3 — Expression of kinases and phosphatases involved in Cofilin phosphorylation/dephosphorylation cycle. Western blotting of phospho LIMK1/LIMK2 and LIMK2 do not show any significant difference in WT and CAP2 mutant brain lysate. The levels of other kinases like TESK1 and ROCK1 were also unaltered in mutant brain lysate as was the level of chronophin. [file Image_3.TIF]
